# 15, 16-Dihydrotanshinone I Inhibits Hemangiomas through Inducing Pro-apoptotic and Anti-angiogenic Mechanisms *in Vitro* and *in Vivo*

**DOI:** 10.3389/fphar.2018.00025

**Published:** 2018-01-30

**Authors:** Yihong Cai, Fan Lv, Nurshat Kaldybayeva, Abilova Zhamilya, Zhixiang Wu, Yeming Wu

**Affiliations:** ^1^Department of Pediatric Surgery, Xinhua Hospital, School of Medicine, Shanghai Jiaotong University, Shanghai, China; ^2^Division of Pediatric Oncology, Shanghai Institute of Pediatric Research, Shanghai, China; ^3^State Key Laboratory of Drug Research and Natural Products Chemistry Department, Shanghai Institute of Materia Medica, Chinese Academy of Sciences, Shanghai, China

**Keywords:** infantile hemangiomas, 15,16-dihydrotanshinone I, propranolol, apoptosis, anti-angiogenesis

## Abstract

Infantile hemangioma (IH) is a common and benign vascular neoplasms, which has a high incidence in children. Although IH is benign, some patients experience complications such as pain, functional impairment, and permanent disfigurement. Treatment options for IH include corticosteroids, surgery, vincristine, interferon or cyclophosphamide. However, none of these modalities are ideal due to restrictions or potential serious side effects. There is thus a great need to explore novel treatments for IH with less side effects. Angiogenesis, vasculogenesis and tumorigenesis are the main features of IH. Tanshen is mostly used in Chinese traditional medicine to treat hematological abnormalities. Therefore, the aim of our study was to evaluate anti-proliferation and anti-angiogenesis effects on hemangiomas cells by extracted Tanshen compounds compared with propranolol, the first-line treatment for IH currently, both *in vitro and in vivo.* Cell viability, apoptosis, protein expression and anti-angiogenesis were analyzed by CCK8, Annexin V staining, Western blot and tube formation, respectively. The anti-tumor activity *in vivo* was evaluated using a mouse xenograft model. Fourteen major compounds extracting from Tanshen were screened for their ability to inhibit hemangiomas cells. Of the 14 compounds investigated, 15,16-Dihydrotanshinone I (DHTS) was the most potent modulator of EOMA cell biology. DHTS could significantly decrease EOMA cells proliferation by inducing cell apoptosis, which is much more efficient than propranolol *in vitro*. DHTS increased the expression of several apoptosis-related proteins, including caspase9, caspase3, PARP, AIF, BAX, cytochrome c, caspase8 and FADD and significantly inhibited angiogenesis, as indicated by reduced tube formation and diminished expression of vascular endothelial cell growth factor receptor 2 and matrix metalloproteinase 9. In nude mice xenograft experiment, DHTS (10 mg/kg) could significantly inhibit the tumor growth of EOMA cells as well as propranolol (40 mg/kg). Our study showed that DHTS was much more effective than propranolol in inhibiting hemangiomas proliferation and angiogenesis *in vitro* and *in vivo*, which could have potential therapeutic applications for treatment of IH.

## Introduction

Infantile hemangiomas (IH) is a common and benign vascular neoplasm with an estimated prevalence of 5–10% (Drolet et al., 2005). IH is most common in female, premature, and low-birth-weight infants (Drolet et al., 2008). These tumors could be solitary or multiple, which undergo rapid growth followed by spontaneous, slow and even incomplete involution for up to 10 years. Although about 80–95% of the tumors of IH could spontaneously involute, a sort of patients suffer from complications resulting in pain, functional impairment, or permanent disfigurement (Chang et al., 2008; Darrow et al., 2015). The pathogenesis of IH has not been fully elucidated. Epidemiological studies and rare familial cases suggest a genetic influence (Haggstrom et al., 2007).

Propranolol—a non-selective beta adrenergic antagonist, which was widely used for hypertension, angina pectoris, myocardial infarction, migraines and so on, had become the first therapeutic choice since its impressive effects on IH were serendipitously discovered in Léautélabrèze et al. (2008). Besides propranolol, the other medical treatment options of IH include corticosteroids, and vincristine, interferon or cyclophosphamide (Ji et al., 2014). However, none of therapeutic modalities are ideal due to restrictions or potentially serious side effects. Propranolol can cause symptomatic bradycardia, hypotension, hypoglycemia, hypoglycemia-induced seizures, and not all IH patients show a response (Frieden and Drolet, 2009). Particularly, the molecular mechanisms of propranolol treatment on IH remain largely unknown (Storch and Hoeger, 2010), which is a potential hazard to the children with IH. Thus, here is a great need for novel therapies, which should be more effective and less side effects as compared propranolol.

Infantile hemangioma was mostly described as “embryonic tissue” or “angiogenic disease” with high expression of genes involved in vasculogenesis, angiogenesis, tumorigenesis and associated signaling pathways (Folkman et al., 1997; Chang et al., 1999; Harbi et al., 2016). Angiogenesis and vasculogenesis are the main features of IH. In hemangioma-derived endothelial cells and hemangioma tissues, vascular endothelial growth factor(VEGF), fetal liver kinase-1(Flk-1)/vascular endothelial growth factor receptor 2(VEGFR-2), VEGFR-1, tunica interna endothelial cell kinase-1 (Tie-1), Tie-2, Insulin-like growth factor-2(IGF-2) and angiopoietin-2 are highly expressed, while reduced in involuting hemangioma (Yu et al., 2004). Furthermore, the proliferation rate and invasion capability of endothelial cells in vascular tumors are much greater as compared to the normal vascular endothelial cells. Once hemangiomas reach their maximum size, they initiate spontaneously regression that is characterized by endothelial apoptosis (Iwata et al., 1996; Razon et al., 1998; Frischer et al., 2004).

Plant-derived phytochemicals have been widely used as therapeutic agents for 1000s of years in China. Herbal extracts are commonly used worldwide for treating illness and improving health. In traditional Chinese medicine, Hemangiomas generally belong to “RouLiu,” which are caused by obstruction of meridian and collateral, and are required to “activate blood circulation”. The dry root of S. miltiorrhiza, also called Tanshen, is widely used in traditional Chinese medicine to “active blood circulation” and to treat hematological abnormalities, such as heart disease, hepatitis, hemorrhage, menstrual abnormalities, and collagen-induced platelet aggregation (Lu et al., 2011). Water-soluble phenolic acids and lipophilic tanshinones are the major extractive components of Tanshen, and they have been shown in many studies to have beneficial effects on vascular diseases, especially atherosclerosis and blood clotting abnormalities (Lu et al., 2011). In addition, lipophilic tanshinones were certificated as an anti-cancer agent, based on their anti-proliferative, anti-angiogeneic and pro-apoptotic activities against a broad spectrum of tumors (Lee et al., 2008; Lin et al., 2013).

Since propranolol is the first line of clinical drug and widely used for IH patients, the new developing drugs should be more effective and less toxicity than propranolol or at least comparable with propranolol with other advantages. To this end, we systematically evaluate the pharmaceutical potential of extracts from Tanshen on the influence of IH growth *in vitro* and *in vivo* as compared to propranolol. Interestingly, we found that DHTS was an effective compound of inhibiting hemangioma cells, which was more potent than propranolol. Furthermore, our data revealed that DHTS could significantly induce cell apoptosis by mitochondrial- and extrinsic- pathways and inhibit angiogenesis both *in vitro* and *in vivo*. Interestingly, DHTS could achieved similar effects at much lower doses as compared to propranolol. Our research may provide a foundation for the clinical development of DHTS as an alternative treatment for IHs.

## Materials and Methods

### Cell Line and Cell Culture

Endothelioma(EOMA) cell line was originally derived from a spontaneously arising hemangioendothelioma in the 129/J strain (Hoak et al., 1971). The cell line was purchased from American Type Culture Collection (ATCC) and was cultured in 1640 medium (Gibco) with 10% fetal bovine serum (Gibco), 100 mg/l penicillin (Sigma) and 100 μg/ml streptomycin at 37°C, 5% CO_2_ and a humidified atmosphere.

### Drugs and Reagents

All extracted compounds of Tanshen were from Shanghai Institute of Materia Medica (Chinese Academy of Sciences); Captisol was derived from MCE (MedChem Express); Caspase 3, Caspase 8, Caspase 9, Aif, PARP, Bax, FADD and Cyst3 were all from Abclonal; FITC-Annexin V and PI were from BD Biosciences (Sandiego); Matrigel was from Corning; CCK kit and crystal violet were from Yeasen (China); Nude mice were provided from Shanghai XinHua Hospital.

### *In Vitro* Cytotoxity

The cytotoxicity of all drugs was measured by cell counting kit-8 (CCK8) (Yeasen, China). These drugs were dissolved by DMSO and stocked at -20°C. Briefly, about 3 × 10^3^ cells per well were plated in 96-well plates, and then were treated with different drugs (dissolved by DMSO) at different concentrations or DMSO. All plates were added with the same concentration of DMSO. After 72 h, the medium containing drugs or DMSO were all replaced with 10% CCK-8 solution. Incubate the plate at 37°C for 1 h.

### Colony Formation Assay

About 1 × 10^3^ cells per well were seeded in six-well plates and treated with DHTS and propranolol. DHTS, propranolol or DMSO (diluent) in various concentrations for 24 or 48 h. Then the fresh medium was added to allow cell growth for 1 week. The colonies with more than 50 cells were counted after staining with crystal violet.

### Cell Apoptosis Analysis

To detect apoptosis, cells were incubated with DHTS, propranolol or DMSO in different concentrations for 48 h. Then cells were harvested, washed twice with cold 1 × PBS, and re-suspended in 200 μL binding buffer at the density of 1 × 10^5^cells/mL. Then cells were stained with 5 μL Annexin-V (BD Biosciences)for 10 min in dark condition at room temperature and then stained with 5 μL PI for 1 h. At last, cells were analyzed by flow cytometry. The early apoptosis was evaluated based on the percentage of cells with Annexin V+/PI-, while the late apoptosis was Annexin V+/PI+. The results were indicated as mean values from three independent determinations.

To visualize apoptotic bodies, EOMA cells were exposed to different concentrations of DHTS for 24 h, fixed in 4% paraformaldehyde and stained with 1 ml 10 μg/ml Hochest 33342 (Sigma) for 30 min at 37°C in the dark. After thoroughly washed with PBS, the cells were checked for karyopyknosis under the inverted fluorescence microscope.

### Western Blot Analysis

The expression levels of different proteins in cells were performed by Western blot analysis. Cells were treated with DHTS, propranolol or DMSO at various concentrations for 48 h. Cells were washed with cold 1 × PBS, collected and lysed with RIPA lysis buffer (Beyotime) for 30 min on ice, then centrifuged at 12,000 *g* at 4°C for 10 min. The concentration of total protein was determined by BCA protein assay kit (Beyotime). Equal amounts (10 μg) of protein samples were subjected to SDS-PAG Electrophoresis and transferred onto polyvinylidene difluoride (PVDF) membranes (Millipore). The blots were blocked in 5% non-fat milk, and incubated with various primary antibodies (1:1000), followed by incubation with secondary antibodies (1:2000) (Yeasen, China) conjugated with horseradish peroxidase. The protein bands were visualized by the chemiluminescent reagents (Millipore). Antibodies to Bax (1:1000, A0207), Aif (1:1000, A2568), Parp (1:1000, A0942), Caspase3 (1:1000, A0214), Caspase8 (1:1000, A0215), Caspase9 (1:1000, A11451), Cyst3 (1:1000, A1561), GAPDH (1:1000, AC001) and FADD (1:1000, A5819) were from Abclonal.

### Immunohistochemistry

After being excised, tumors were fixed with 4% paraformaldehyde and embedded with paraffin. Primary antibodies against CD34, MMP9, VEGFR2 and Caspase 3 were obtained from Abclonal. Slides were stained with primary antibodies, then washed, and stained with secondary antibody. Some sections were stained with H&E for the histological analysis. The stained sections were observed by the Leica CTR6000 microscope at a magnification of ×400.

### Tube Formation

Unpolymerized Matrigel (Corning) was placed in a 96-well plate at 10 μl/well and polymerized for 1 h at 37°C. EOMA cells (3 × 10^4^cells/well) in 50 μl medium, as well as in the presence or absence of DI (0, 1.25, 2.5 μM) or propranolol (0, 50, 100 μM) were layered onto the Matrigel surface. After 2h, 4h of incubation, cell growth and 3D organization were observed under a microscope.

### Xenograft Model Assay

Female BALB/c nude mice at age 4–6 weeks were from Experimental Animal Centre of Shanghai XinHua Hospital and kept in a specific pathogen-free facility. Mice were inoculated with EOMA cells (1 × 10^7^suspended in 0.2 mL PBS for each mouse) in the axilla. After reaching an average tumor volume of 100 mm^3^, the animals were randomized into different groups that were treated intraperitoneally with DHTS (10mg/kg) (Liu et al., 2015), propranolol (40 mg/kg) (Li et al., 2015), or vehicle control (captisol containing 20% DMSO) thereafter. Measurement of tumor growth with a digital caliper was made three times a week. Tumor volumes were calculated by the following formula: V = L × W^2^/2 (V is the volume, L is the length, and W is the width). At the end of experiment, the mice were sacrificed, and the tumors were weighed and dissected. Tumors were fixed with 4% paraformaldehyde and embedded in paraffin for IHC staining. The animal experimental protocols were approved by the Animal Ethics Committee of Xinhua Hospital affiliated to Shanghai Jiao Tong University.

### Statistics Analysis

Data were calculated using SPSS and expressed as mean ± SE. IC_50_ was calculated by a non-linear regression model with a sigmoidal dose-response curve. Comparisons between controls and treated groups were determined by paired *t-*test or one-way ANOVA followed by Tukey’s multiple comparison tests. The significant difference was considered at the level of *p* < 0.05.

## Results

### DHTS Exhibit Potent Anti-proliferative Activity

**FIGURE 1:**
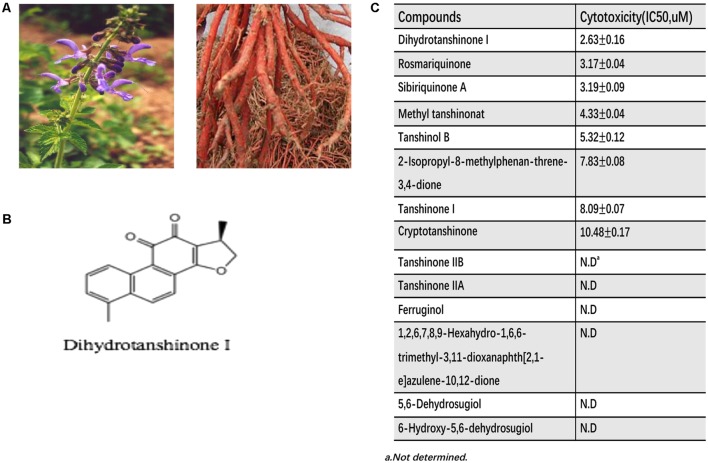
The cytotoxity of extracted fourteen compounds of Tanshen on EOMA. **(A)** The pictures of Tanshen. **(B)** Chemical structures of Dihydrotanshinone I. **(C)** Half inhibition concentration (IC_50_) of extracted Tanshen on EOMA cells.

**FIGURE 2:**
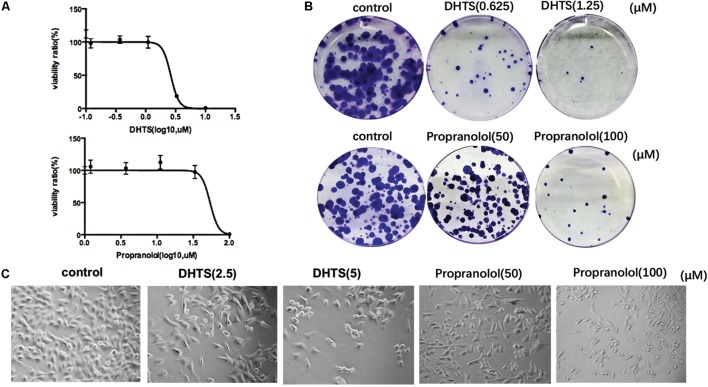
15,16-Dihydrotanshinone I (DHTS) and propranolol inhibited cell proliferation. **(A)** DHTS inhibited the proliferation of EOMA cell lines dose-dependently as well as propranolol. Cell viability determined by CCK8 assay. IC_50_ value treated by DHTS was 2.63 ± 0.16 μM, while 53.6 ± 1.73 μM by propranolol. **(B)** Images of cell colonies after treatment with different concentrations of DHTS or propranolol for 24 h. **(C)** Morphologic characteristics of EOMA cells which treated by DHTS, propranolol or DMSO (control) were observed by microscopy.

To assess whether Tanshen contains hemangiomas-inhibitory substances, we tested the effect of fourteen main compounds of Tanshen on the EOMA cells, a well-established model for IH study (Gordillo et al., 2002). EOMA cells were exposed to the selected 14 compounds of Tanshen for 72 h, and the cell proliferation was determined by CCK8 assay. As shown in **Figure 1**, DHTS was found to be the most potent extract, with an IC_50_ = 2.63 ± 0.16 μM. As control, we also investigated the anti-proliferative effects of propranolol (0, 1.8, 3.7, 11, 33, and 100 μM) on EOMA cells. As shown in **Figure 2A**, DHTS was much more effective than propranolol.

We next examined colony formation assays to further determine DHTS inhibitory effects on EOMA cells. The results showed that the colony formation ability cells were significantly reduced by DHTS. The same effect was also observed in the cells treated by propranolol, but with almost 20-fold of drug concentration as compared with DHTS (**Figure 2B**). As compared with control, the morphology of cells treated with DHTS or propranolol significantly turned round and the rate of proliferation was also decreased. (**Figure 2C**).

### DHTS Induced IH Cells Mitochondria- and Fas-Mediated Apoptosis

**FIGURE 3:**
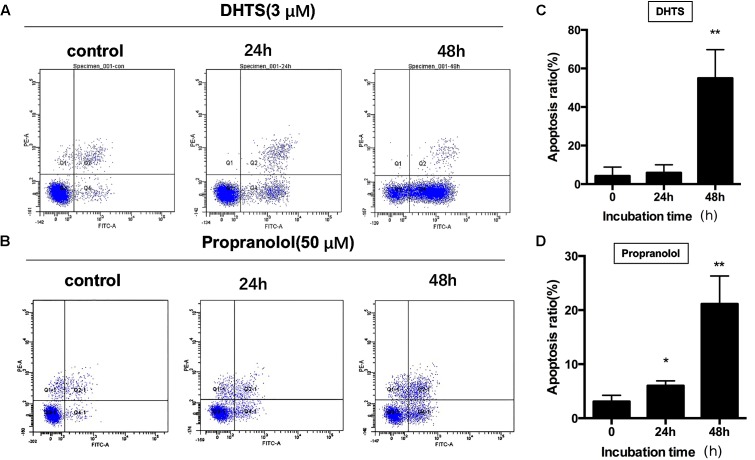
15,16-Dihydrotanshinone I and propranolol induced time-dependent apoptosis in EOMA cells. **(A,B)** EOMA cells were treated with 2.5 μM DHTS and 50 μM propranolol for 0, 24, or 48 h. Cells were collected and were detected with flow cytometry analysis. **(C,D)** The quantitative data showed the percentage of apoptotic cells in **(A,B)**. ^∗^*P* < 0.05, ^∗∗^*P* < 0.01 compared with drug-untreated group.

**FIGURE 4:**
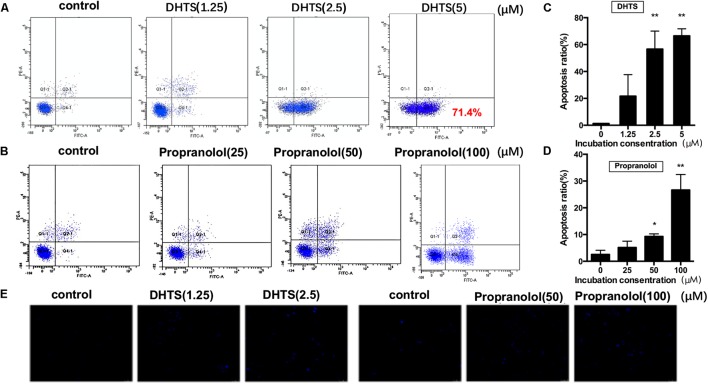
15,16-Dihydrotanshinone I and propranolol induced dose-dependent apoptosis in EOMA cells. **(A,B)** EOMA cells were treated with different concentrations of propranolol (0, 25, 50, and 100 μM) and DHTS (0, 1.25, 2.5, and 5 μM). Cells were collected and were detected with flow cytometry analysis. **(C,D)** The quantitative data showed the percentage of apoptotic cells in A and B. ^∗^*P* < 0.05, ^∗∗^*P* < 0.01 compared with drug-untreated group; **(E)** The Hochest33342 staining assay revealed that DHTS and propranolol facilitated cell apoptosis in EOMA cells.

To further investigate the potential biological effects of DHTS on EOMA cells, we examined the apoptosis of EOMA cells after treatment. Exposing EOMA cells to either DHTS(3 μM) or propranolol(50 μM) for different time suggesting that the percentage of early apoptotic cells portion (Annexin V-FITC positive and PI negative), was significantly increased in a time-dependent manner (**Figures 3A–D**). Then, we treated cells with different concentration of DHTS (0, 1.25, 2.5, 5 μM) and propranolol (0, 50, 100 μM) for 48 h. The percentage of early apoptotic cells portion of DHTS and propranolol treatment were both dose-dependently increased with the raising concentration (**Figures 4A–D**). While early apoptotic cells were much more generated by DHTS treatment than propranolol at significantly lower doses. Consistently, the apoptotic cells induced by DHTS and propranolol treatment were further confirmed by Hochest33342, a fluorescent DNA binding dye for apoptosis cells (**Figure 4E**).

**FIGURE 5:**
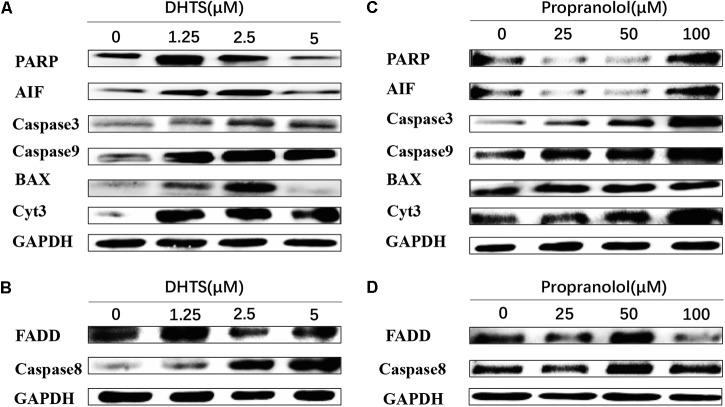
Apoptosis related proteins were detected in EOMA cells after treated with DHTS and propranolol. **(A,B)** DHTS induced PARP, Aif, Caspase9, Caspase3, Bax and Cyts3 in low concentration more than in high concentration, while induced FADD and Caspase 8 more in relatively high concentration. **(C,D)** Propranolol induced PARP, Aif, Caspase9, Caspase3, Bax and Cyts3 in high concentration more than in low concentration, while induced FADD and Caspase 8 more in relatively low concentration.

We next investigated the potential signaling pathways via which the DHTS induced apoptosis in IH. Our data suggested that treatment with DHTS, particularly at low concentrations, significantly increased the expression of Bax, poly (ADP-ribose) polymerase (PARP), Caspase-9, Caspase-3, apoptosis-inducing factor (AIF) and cytochrome c. In contrast, treatment of EOMA cells with higher DHTS concentrations more induced the expression of Fas-mediated apoptosis-associated proteins, such as FADD and caspase-8 (**Figures 5A,B**). Compared with DHTS, propranolol had the opposite effect to DHTS: Fas-mediated apoptosis was induced by low propranolol concentrations and the mitochondrial pathway was activated at higher concentrations (**Figures 5C,D**).

### DHTS Inhibits Angiogenesis *in Vitro*

**FIGURE 6:**
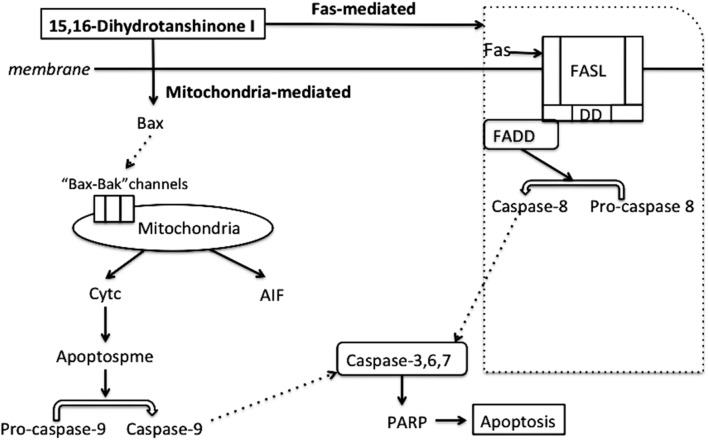
Schematic diagram of DHTS-induced mitochondria- and Fas-mediated apoptosis.

**FIGURE 7:**
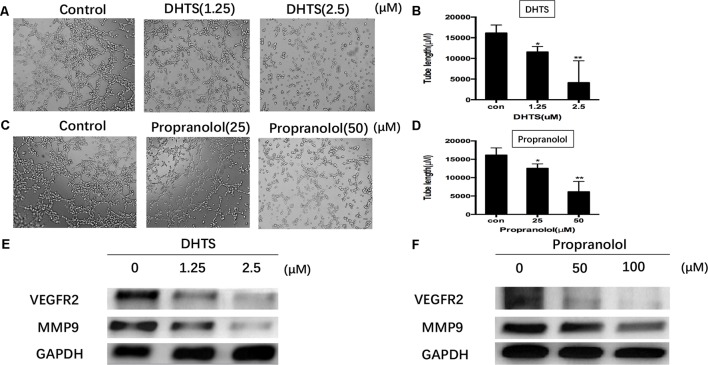
Effects of DHTS and propranolol on tube formation of EOMA cells. **(A,B)** Cell cultured on Matrigel, treated by DHTS with different concentrations (0, 1.25, and 2.5 μM) after 4 h and then photographed under a microscope. ^∗^*P <* 0.05, ^∗∗^*P* < 0.01 compared with normal group. **(C,D)** Cell cultured on Matrigel and treated by propranolol (0, 25, and 50 μM) with different concentrations after 4h. ^∗^*P* < 0.05, ^∗∗^ *P* < 0.01 compared with normal group. **(E,F)** VEGFR2 and MMP9 were both down-regulated by DHTS and propranolol treatment.

The production of tubular structures is an important step in angiogenesis (Khoo et al., 2011). We thus investigated the effects of DHTS on hemangiomas tube formation in *vitro*. As shown in **Figure 6**, EOMA cells plated on Matrigel and incubated with control medium aligned to form lumen-like structures and anastomotic tubes with multi-centric junctions. When exposing to either DHTS or propranolol for 24 h, the EOMA cells formed fewer tubes, as well as fewer and weaker anastomoses, in a dose-dependent manner. In addition, our data suggested that DHTS showed 10 times more potent on tube formation of EOMA cells as compared to propranolol. VEGF/VEGFR2-mediated signaling is crucial for angiogenesis (Khoo et al., 2011). As expected, the expression levels of VEGFR2 and MMP-9 were significantly down-regulated after exposing to both DHTS and propranolol (**Figures 7E,F**). Together, our data clearly illustrated that DHTS could inhibit hemangiomas angiogenesis with a much more potency than propranolol.

### DHTS Inhibits the Growth and Angiogenesis of Hemangiomas *in Vivo*

To extend the observation made in cultured cells and to assess the efficacy of DHTS *in vivo*, we made the subcutaneous xenograft tumor models by transplanting EOMA cells into nude mice. As shown in **Figure 7A**, either DHTS (10 mg/kg per 3 days) or propranolol (40 mg/kg per 3 days) treatment as a single agent resulted in significant tumor volume reduction compared with control group. The mean body weights at the end of the experiment were similar for all groups, suggesting that DHTS had few toxic effects. These results *in vivo* demonstrated that DHTS exhibited anti-tumor activities *in vivo*. After the treatment of DHTS, propranolol and dissolvent *in vivo*, VEGFR2, MMP-9, CD34 and Caspase 3 were detected among different groups by immunohistochemistry. CD34, VEGFR2 and MMP-9 were down-regulated, while Caspase 3 was significantly up-regulated after treatment (**Figure 7C**). These results demonstrate that DHTS has anti-IHs activity by inhibiting angiogenesis and inducing cell apoptosis.

## Discussion

In this study, we examined the effects of the main extracted compounds of Tanshen on EOMA cells, and we identified DHTS as the most potently cytotoxic of all compounds examined. DHTS exhibits both cytotoxicity and anti-angiogenic activity in hemangiomas cells, which is much more effective than the first-line clinical drug for IH—propranolol both *in vitro* and *in vivo*. DHTS was previously reported to treat cardiovascular disease, hepatitis, inflammation, and cancer (Zhou et al., 2005; Wang et al., 2007). DHTS could increase ATF3 expression contributing to DHTS-induced apoptosis in both non-malignant SW480 and malignant SW620 colorectal cancer cells (Suk et al., 2013). The ROS- and MAPK-dependent pathways appear to be involved in the signaling for DHTS-induced apoptosis in colon cancer cells (Lee et al., 2009; Wang et al., 2013). In SMMC-7721 cells, DHTS could reduce angiogenesis by blocking VEGF-mediated signal transduction.

Although propranolol has become the first line of clinical drug and widely used for IH patients, more and more side effects of propranolol have been reported (Frieden and Drolet, 2009). There is a great need to develop new drugs for IH patients, which should be more potent and less toxicity than propranolol or at least comparable potent as propranolol but with other advantages. To this end, we set propranolol as positive control group for the following experiment. In line with data reported previously (Albiñana et al., 2012, 2015), the IC_50_ of propranolol treatment on EOMA cells was 53.6 ± 0.16 μM. While, parallel experiment suggested DHTS had a much higher potency than propranolol (IC_50_ = 2.63 ± 0.16). To evaluate the biological effects of the drugs, we performed the cell flow cytometer analysis. The result showed that the early apoptosis but not necrosis was significantly increased after exposing to either high concentration of DHTS or propranolol, suggesting that the EOMA cell inhibition by DHTS and propranolol was induced by chemo-effect rather than toxicity. Apoptosis and angiogenesis were two major events in IH and also potential mechanisms of reducing tumor growth (Kroemer and Martin, 2005), we focused our following attention on the effects of DHTS and propranolol on apoptotic and angiogenic signaling in EOMA cells.

Apoptosis, or programmed cell death, plays an important role in hemeostasis, tissue development and diseases (Elmore, 2007). There are mainly three apoptotic pathways: the death receptor (extrinsic) pathway, the mitochondrial pathway and endoplasmic reticulum stress-mediated apoptosis. The Fas-mediated apoptosis pathway is initiated by Fas ligand binding to Fas, which activates the caspase-8 pathway by directly recruiting FADD (Workman and Habelhah, 2003). Activated caspase-8 could active caspase-3, 6 and 7 (Lavrik and Krammer, 2012). Our study showed that FADD and caspase-8 in EOMA cells were both increased by DHTS and propranolol treatment, consistent with induction of extrinsic apoptosis pathway. Interestingly, DHTS induced extrinsic apoptosis more effectively at high concentrations, whereas the opposite trend was observed with propranolol (**Figure 5**). The mitochondrial apoptotic pathway is activated by cellular damage, such as that induced by ionizing radiation, chemotherapeutic drugs, and deprivation of cytokines and other essential factors (Fan et al., 2005; Elmore, 2007; Martinou and Youle, 2011). Briefly, all stimuli lead to changes in the inner mitochondrial membrane resulting in the destroyed mitochondrial permeability transition pore, and then release pro-apoptotic proteins from intermembrane into the cytosol, which concludes cytochrome c, smac, AIF and so on (Renault and Manon, 2011; Zhang et al., 2011). In our *in vitro* studies, DHTS induced the expression of Bax, PARP, Aif, Caspase9, Caspase3 and cytochrome c, with DHTS having a greater effect than propranolol (**Figure 5**). In contrast to the effects of DHTS on the extrinsic apoptosis pathway, mitochondrial apoptosis was induced by low, but not high, concentrations of DHTS. However, here too, the reverse was true for propranolol. The detailed biological mechanisms underlying this observation remains unclear.

Hemangiomas was described as a relatively form of angiogenesis (Frischer et al., 2004). Manipulating EOMA cells both *in vitro* and *in vivo* provides an efficient model to study the influence of tumor cell derived signals that regulate angiogenesis through the recruitment of host endothelial cells (Atalay et al., 2003). VEGF/VEGFR2-induced signaling is a key step for tumor angiogenesis and its inhibition is an effective therapeutic modality to prevent cancer growth and metastasis (Goel and Mercurio, 2013). The anti-angiogenesis capacity could be detected by tube formation *in vitro*. Here, we found that DHTS and propranolol possessed the capacity of anti-angiogenesis by suppressing tube formation (**Figure 7**). *In vivo*, the tumors expression of MMP9 and VEGFR2, which are the main hallmarks of angiogenesis, was obviously decreased by DHTS and propranolol treatment. DHTS showed anti-angiogenesis capacity under much lower concentration than propranolol.

**FIGURE 8:**
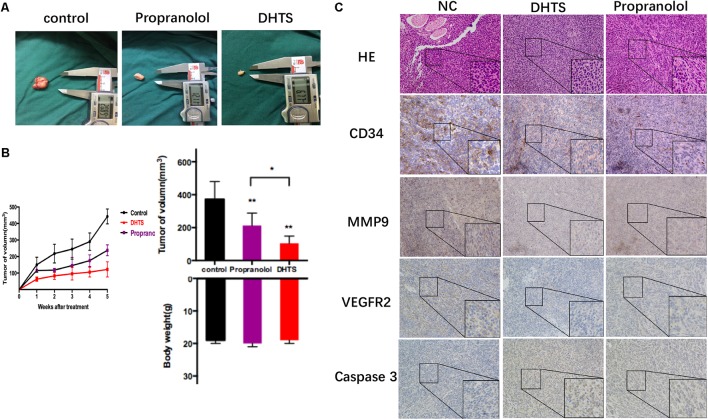
15,16-Dihydrotanshinone I and propranolol inhibits tumor growth *in vivo*. **(A)** The tumors were excised from mice incubated EOMA cells after treatment and measured by digital caliper. Propranolol and DHTS inhibited tumor growth after treatment. **(B)** Tumor volumes were measured by digital caliper three times a week. Figures showed tumor and body weights of mice after final treatment. ^∗∗^*P <* 0.01 (One-Way ANOVA was used for the data analysis) compared with control group, ^∗^*P*>0.05, The plotted error bars represent mean ± SEM. **(C)** CD34, MMP9, VEGFR2 and Caspase3 were detected by Immunohistochemistry after xenograft tumor tissues were excised.

Our results showed that both compounds inhibited the growth of hemangioma cells both *in vitro* and *in vivo*. Although propranolol is the first-line treatment of IH, our data illustrated that DHTS was considerably more potent than propranolol in eliciting anti-angiogenic and pro-apoptotic activity in hemangioma cells. In the *in vivo* study, both compounds inhibited xenograft hemangioma growth without inducing major side effects, which provide the possibility to develop a new drug on IH (**Figure 8**). Our data implies DHTS as a high potent compound for anti-hemangioma cells, but further understanding of the signaling pathways by which DHTS and propranolol execute the pro-apoptotic and anti-angiogenic pathways and additional experiments on human patient-derived xenografts (PDX) will both be beneficial for developing DHTS as an anti-hemangiomas drug in the further.

## Conclusion

The aim of the present study was to evaluate the potential therapeutic effect of DHTS on IHs. Our findings reveal for the first time that *in vivo* and *in vitro*, the anti-angiogenic and apoptosis-inducible activity of DHTS occur at significantly lower doses than propranolol, which could be potentially become a new and high effective compound for the treatment of IHs.

## Author Contributions

YC mainly carried out the experiments, executed the work and drafted the manuscript. FL participated in most of *in vitro* and *in vivo* experiments. NK and AZ separated extracted compounds from Tanshen. ZW and YW improved the design, drafted of this work and contributed to the revision of this manuscript. All listed authors approved the version for publication, and agreed to be accountable for all aspects of this work.

## Conflict of Interest Statement

The authors declare that the research was conducted in the absence of any commercial or financial relationships that could be construed as a potential conflict of interest.
